# Organ Sparing for Locally Advanced Rectal Cancer after Neoadjuvant Treatment Followed by Electrochemotherapy

**DOI:** 10.3390/cancers13133199

**Published:** 2021-06-26

**Authors:** Daniela Rega, Vincenza Granata, Antonella Petrillo, Ugo Pace, Cinzia Sassaroli, Massimiliano Di Marzo, Carmela Cervone, Roberta Fusco, Valeria D’Alessio, Guglielmo Nasti, Carmela Romano, Antonio Avallone, Biagio Pecori, Gerardo Botti, Fabiana Tatangelo, Piera Maiolino, Paolo Delrio

**Affiliations:** 1 Division of Colorectal Surgery, Istituto Nazionale Tumori IRCCS Fondazione Pascale—IRCCS di Napoli, I-80131 Naples, Italy; d.rega@istitutotumori.na.it (D.R.); u.pace@istitutotumori.na.it (U.P.); c.sassaroli@istitutotumori.na.it (C.S.); m.dimarzo@istitutotumori.na.it (M.D.M.); carmela.cervone@istitutotumori.na.it (C.C.); p.delrio@istitutotumori.na.it (P.D.); 2 Division of Radiology, Istituto Nazionale Tumori IRCCS Fondazione Pascale—IRCCS di Napoli, I-80131 Naples, Italy; a.petrillo@istitutotumori.na.it; 3 IGEA SpA Medical Division-Oncology, Via Casarea 65, Casalnuovo di Napoli, I-80013 Napoli, Italy; r.fusco@igeamedical.com (R.F.); v.dalessio@igeamedical.com (V.D.); 4 Division of Abdominal Medical Oncology, Istituto Nazionale Tumori IRCCS Fondazione Pascale—IRCCS di Napoli, I-80131 Naples, Italy; g.nasti@istitutotumori.na.it (G.N.); c.romano@istitutotumori.na.it (C.R.); a.avallone@istitutotumori.na.it (A.A.); 5 Division of Abdominal Radiotherapy, Istituto Nazionale Tumori IRCCS Fondazione Pascale—IRCCS di Napoli, I-80131 Naples, Italy; b.pecori@istitutotumori.na.it; 6 Division of Pathological Anatomy, Istituto Nazionale Tumori IRCCS Fondazione Pascale—IRCCS di Napoli, I-80131 Naples, Italy; g.botti@istitutotumori.na.it (G.B.); f.tatangelo@istitutotumori.na.it (F.T.); 7 Division of Pharmacy, Istituto Nazionale Tumori IRCCS Fondazione Pascale—IRCCS di Napoli, I-80131 Naples, Italy; p.maiolino@istitutotumori.na.it

**Keywords:** electrochemotherapy, locally advanced rectal cancer, endoluminal electrodes, conservative surgery, total mesorectal excision

## Abstract

**Simple Summary:**

This is a Phase II randomized controlled trial conducted with the aim of investigating whether the use of Electrochemotherapy after neoadjuvant therapy (ECT) and before surgery in patients with major clinical response allows for a more conservative surgical approach in patients with Locally Advanced Rectal Cancer (LARC) in comparison with the control group that will not receive ECT. The treatment response, in both the control arm and in the treatment arm, will be assessed using the histopathological tumor regression grade on tissue specimens after local excision.

**Abstract:**

Background: Currently, 45–55% of rectal cancer patients receive preoperative chemo- radio-therapy for Locally Advanced Rectal Cancer (LARC). The idea of our study is to use Electrochemotherapy (ECT) before surgery, in patients with major clinical response after neoadjuvant therapy, to allow for a more conservative surgical approach. Objective: To evaluate the increase of the complete response rate after neoadjuvant treatment in LARC and to spare organ function due to total mesorectal excision (TME). Patients and Methods: This is a Phase II randomized controlled trial enrolling 70 patients that will be developed in two stages. In the first step, 28 patients will be enrolled: 14 of these will receive ECT for four weeks after neo-adjuvant treatment and then local excision (treatment group) and 14 patients will receive neo-adjuvant treatment and then local excision (control group). If an increase of response rate is observed in the first stage, and/or feasibility/safety is demonstrated, the second stage of the trial will be performed, enrolling an additional 42 patients. The treatment response. in both the control arm and the treatment arm, will be assessed using the histopathological tumor regression grade on tissue specimens after local excision.

## 1. Introduction

Electroporation (EP) is a technique that is able to increase the permeability of the cell membrane by applying a short and intense electrical field that allows molecules that are not, or barely, permeable, to enter into the cell. Electrochemotherapy (ECT) is the combination of a cytotoxic drug and EP.

After the first electrochemotherapy clinical trials [1,2,3,4], the Standard Operating Procedures for electrochemotherapy with the CLINIPORATOR™ were defined [4,5]. Other studies were carried out that confirmed the clinical effectiveness of ECT and its acceptable safety profile [4,6,7,8,9], as well as that it had significantly higher effectiveness than bleomycin or cisplatin alone [9]. No electrochemotherapy-related serious adverse events were observed [8].

Recently, the feasibility and benefits of ECT have also been observed and documented in deep solid tumors, such as liver metastasis, primitive liver tumors (cholangiocarcinoma and hepatocellular carcinoma) and pancreatic tumors, both in preclinical and clinical studies [10,11,12,13,14,15,16,17,18,19,20,21,22,23,24,25,26], overcoming the difficulty of obtaining the complete electroporation of target lesions. Preliminary results show that the treatment of metastases localized in deep-seated organs is feasible and safe, that ECT is effective in the resolution or regression of tumor nodules, improving patients’ quality of life. Neither acute (intraoperative) nor postoperative serious adverse events, nor clinically significant electrocardiographic, hemodynamic, or serum biologic changes were noted. Amylase or lipase levels were normal in the patients and no bleeding or damage to surrounding viscera occurred [20,21].

Endoluminal allows the endoscopic treatment of colorectal cancer and gastric and esophageal tumors. This device uses a vacuum to capture tumor tissue while an electroporation pulse is applied. Recently, a phase I clinical trial [27] on endoscopic electrochemotherapy for advanced esophageal cancer—the first in humans—has been conducted in six patients using intravenous bleomycin. Treatments were well tolerated, with the main AEs being nausea, vomiting, oral thrush, pneumonia, retrosternal pain, fever, and hoarseness. No serious complications were observed. Five patients had a visual tumor response confirmed by gastroscopy. In two cases, these findings were confirmed with 18F-fluorodeoxyglucose positron emission tomography/magnetic resonance imaging (18F-FDG PET/MRI) as it revealed a reduction of total tumor mass [27].

A phase II clinical trial in non-surgical patients with colorectal cancer is currently ongoing (https://clinicaltrials.gov/ct2/show/NCT03040180, accessed on 10 April 2021). The patients are being treated with bleomycin i.v. according to the ESOPE (European Standard Operating Procedures of Electrochemotherapy) protocol and, in preliminary results, no adverse events or complications have been registered.

Therefore, an endoscopic treatment for colorectal cancer has been investigated and the preliminary results demonstrate that the entire procedure is minimally invasive and completely ambulatory.

## 2. Trial Design

**Figure 1:**
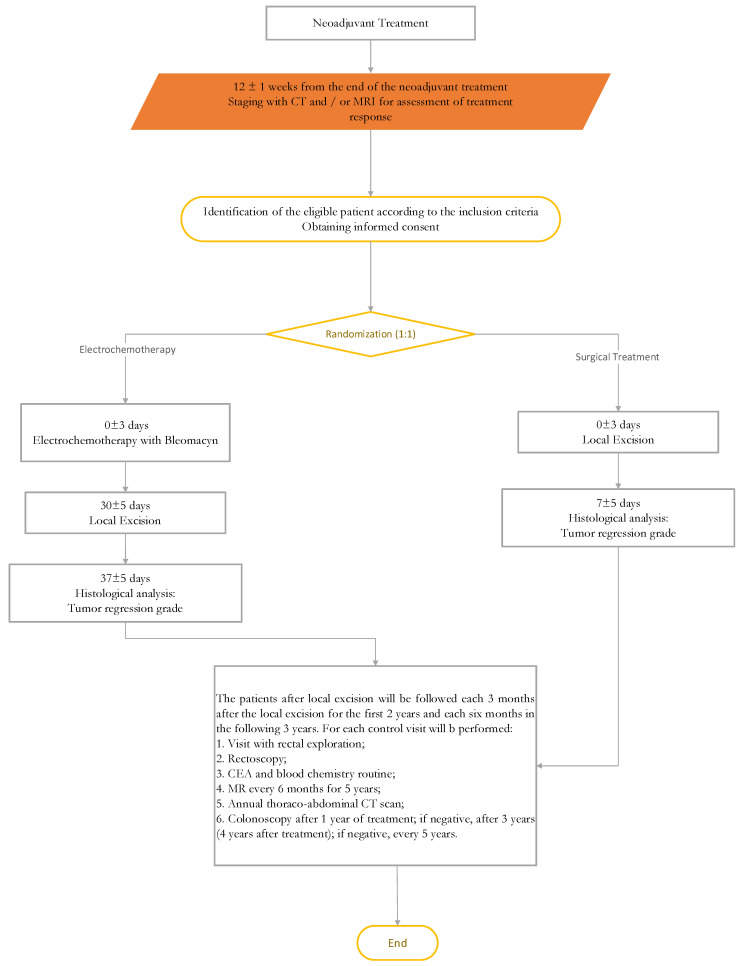
Trial design flowchart.

Seventy patients will be enrolled in a Phase II randomized controlled trial. The study will be developed in two stages. In the first step, 28 patients will be enrolled: 14 of these will receive ECT for four weeks after neo-adjuvant treatment and then local excision (treatment group) and 14 patients will receive neo-adjuvant treatment and then local excision (control group). Electrochemotherapy will be performed with the CLINIPORATOR™ and an intravenous dose of Bleomycin. If an increase of response rate is observed in the first stage and/or feasibility/safety is demonstrated, the second stage of the trial will be performed. In the second step, an additional 42 patients will be enrolled and treated. Trial design flow-chart is showed in Figure 1.

### 2.1. Objectives

#### 2.1.1. Primary Endpoints

1. To assess electrochemotherapy feasibility/safety.

2. To evaluate the complete response rate increase with the aim of organ sparing.

3. Histopathological analysis evaluation will be based on the tumor regression grade (TRG) by Mandard et al. [28], performed on tissue specimens by local excision.

#### 2.1.2. Secondary Endpoints

To evaluate the local recurrence rate, pain control, quality of life and electrochemotherapy toxicity.

### 2.2. Subject Selection

Eligible patients will be randomly assigned (1:1) to the treatment group or the control group. The treatment group assignment will be assigned blindly and sequentially in the order in which eligible patients are enrolled in the study. Patients eligible for inclusion in the protocol (see Table 1) will be informed in detail about the study and, if they consent to participate, they will sign the informed consent form (Appendix A), witnessed by a member of the research group.

### 2.3. Subjects Treatment

Both the Control group and the Treatment group will receive neoadjuvant treatment, either long course radiotherapy with concomitant chemotherapy or short course radiotherapy, before local excision (LE). Long course radiotherapy consists of 45–50 Gy (in fractions of 1.8–2 Gy), with concurrent 5-fluorouracil. Standard fractions of 1.8 Gy/day to the reference point will be given five times a week up to a total dose of 50.4 Gy in 28 fractions [29]. Each subject will receive the standard treatment with capecitabine at a dose of 825 mg/m^2^ twice daily, five days a week, for five weeks.

Short course radiotherapy consists of a dose of 25 Gy in five fractions over one week [29].

External radiation therapy will be performed using a 3-field (one posterior–anterior and two lateral fields) or IMRT (Intensity Modulated Radiation Therapy) technique.

#### 2.3.1. Control Group Treatment

Patients undergoing neoadjuvant treatment for LARC with a major clinical response at restaging will undergo a local excision 16 (±1) weeks after the end of neoadjuvant therapy and histopathological examination with TRG evaluation. Local excision can be performed either with the conventional technique (TAE), or the Transanal endoscopic microsurgery (TEM) or other techniques (TAMIS), provided that the following principles are respected: free macroscopic margin of at least 0.5 cm, full thickness excision, fixation of the operative piece on support to facilitate the histological interpretation of the pathologist.

After histopathological examination, based on pathological findings, Total Mesorectal Excision (TME) can then be planned for the patients if, as expected from clinical practice, ypT ≥ 2, there is the presence of positive margins, TRG > 2 and the cancer is poorly differentiated (G3).

#### 2.3.2. Experimental Group Treatment

In the treatment arm, patients undergoing neoadjuvant treatment for LARC with major clinical response at restaging will undergo Electrochemotherapy (ECT) 12 (±1) weeks after the end of neoadjuvant therapy. After four weeks (±5 days) of ECT, the patients will undergo a surgical treatment with local excision and histopathological examination with TRG evaluation.

After histopathological examination, based on pathological findings, TME can be planned for the patients.

Patients will receive Bleomycin intravenously (15,000 IU BLM/m^2^) and after 8 min, an electric pulse delivered to the lesion will be performed with the CLINIPORATOR™ (IGEA Ltd., Modena, Italy) with the insertion of appropriate CE certificate electrodes for endoluminal treatment [4]. The procedure must be completed within 40 min from the end of the bleomycin injection. The anesthesia will be induced after bleomycin injection and before the application of the electrodes in the case of local anesthesia, or before bleomycin injection in the case of general anesthesia.

### 2.4. Duration of the Study and Follow up

The study will last 36 months with five follow-up years for all patients enrolled. The time for enrolment will be 36 months.

Patients after local excision will be visited every three months in the first two years, and then every six months in the following three years.

This study is subject to stopping rules. Patients can decide to interrupt the treatment. Subjects who discontinue/withdraw from study participation will be evaluated by study personnel.

Starting from the informed consent signature, any event occurring during the treatment phase and/or the follow up phase, will be considered an Adverse Event (AE) and will be recorded. Any clinical or biological abnormalities not present at the trial start will be considered intercurrent events.

The visits scheme is summarized in Table 2.

### 2.5. Description of Study Procedures

Quality of life (QoL), as measured by the EQ5d questionnaire, will be completed at Visits 1, 2, 3, 5, 6, 7, 8, 9, 10, 11 and 12 and will be considered optional from visit 13 to 18. The EQ-5D was developed in Europe and it is a descriptive system of preference-based Health Related Quality Life [30]. The EQ-5D questionnaire also includes a VAS, by which respondents can report their perceived health status with a grade ranging from 0 (the worst possible health status) to 100 (the best possible health status). Pain will be assessed at Visits 1, 2, 3, 5, 6, 7, 8, 9, 10, 11 and 12.

#### 2.5.1. Visit 1

All patients will undergo the same enrollment/baseline visit (Visit 1) procedures. Clinical examination will include the measurement of weight, height and vital signs (pulse, blood pressure). Patients must sign the informed consent form. The Screening/Enrollment Visit (Visit 1) procedures must be performed not more than 12 (±1) weeks after the end of neoadjuvant therapy. Patients who fulfill all inclusion criteria will be enrolled. Patients who are Eastern Cooperative Oncology Group ECOG (ECOG) Performance Status grade 0, 1 or 2 are eligible for study participation [31].

Study procedures at Visit 1 consist of the following: screening against inclusion/exclusion; randomization to ECT or standard treatment; and Complete Blood Count (CBC) and coagulation profiles evaluation. An absolute neutrophil count below 1000/mL, a platelet count below 70,000/mL, and/or an international normalized ratio (INR) above 1.5 will be considered exclusion criteria. The patients will be subjected to rectal exploration; rectoscopy; routine hematochemistry and carcinoembryonic antigen (CEA), Magnetic Resonance (MR) with contrast medium (cm) and Computed Tomography (CT) with contrast medium. Medical and Oncology history, including any previous treatments, concomitant treatments, medications, and events will be registered. Pain will be evaluated according to the Visual Analog Scale (VAS) scale and Quality of Life will be monitored according to the EQ5d questionnaire.

All patients will undergo a pre-anesthetic visit and will be excluded from study participation if not eligible for general anesthesia or monitored anesthesia care (conscious sedation).

The major clinical response after neoadjuvant treatment will be evaluated 12 weeks from the end of radiotherapy with the following tests: laboratory exams, CEA dosage, CT of chest and abdomen with contrast medium, MR pelvis with contrast medium and rigid rectoscopy. The major clinical response is classified as the absence of palpable mass to the digital exploration of the rectum and pathological lymph nodes (>5 mm of diameter along the short axis) to the pelvic MRI, and the absence of deep or superficial ulcers > 2 cm of diameter, at rectoscopy.

#### 2.5.2. Visit 2

The patients with confirmed eligibility will be treated with ECT. The type of anesthesia will be determined by the anesthesiologist. The patients will undergo clinical evaluation, pain assessment by VAS score and blood samples and CEA dosage. The use of concomitant treatment or drug for pain control will be registered.

#### 2.5.3. Visit 3

At day 30 ± 3 (Visit 3), all patients randomized in the control or treatment group will be subjected to LE according to established clinical and institutional practices. The patients will undergo the same procedures as above and according to timing reported in Table 2: Evaluation of routine hematochemistry and CEA, pain assessment by VAS scale and Quality of Life (life quality evaluation according to EQ5d questionnaire) will be completed.

#### 2.5.4. Visit 4

The patients will undergo histological examination; surgical specimens containing the tumor will be evaluated and scored according to TRG, as proposed by Mandard et al. [28], by an expert pathologist who is not aware of the clinical and diagnostic findings. Adverse events/complication will be recorded. Pain evaluation and QoL will be assessed.

#### 2.5.5. Visit 5, 7, 9, 11

After clinical evaluation, the patients will be submitted to rectoscopy, colonoscopy, pain assessment by VAS score and blood samples and CEA dosage. The use of concomitant treatment or drugs for pain control will be recorded. Adverse events/complication will be noted.

#### 2.5.6. Visit 6, 10, 13, 15, 17

After clinical evaluation, the patients will be submitted to rectoscopy, colonoscopy, MRI, pain assessment, blood samples and CEA dosage as above. The use of concomitant treatment or drugs for pain control will be recorded. Adverse events/complication will be noted.

#### 2.5.7. Visit 8, 12, 14, 16, 18

After clinical evaluation, the patients will be submitted to rectoscopy, colonoscopy, CT, MRI, clinical evaluation, pain assessment, blood samples and CEA dosage as above. The use of concomitant treatment or drugs for pain control will be recorded. Adverse events/complication will be noted.

### 2.6. Assessment of Efficacy

The response of treatment in both the control arm and the treatment arm will be assessed with the histopathological TRG on tissue specimens after local excision.

The total number of cases with TRG 1 and 2 [28] will be recorded and the proportion of responses will be compared between the two groups.

If the histological examination documents the presence of carcinoma with at least one of the following characteristics: 1. ypT ≥ 2; 2. Positive margins; 3. TRG > 2; 4. Poorly differentiated form (G3), the local excision will be considered inadequate treatment and the patient will undergo TME.

### 2.7. Assessment of Feasibility/Safety

Any Adverse Event (AE) will be recorded on the case report form as well as the intercurrent events. Subjects will be examined at each visit or if the subject reports any unusual symptoms. The numbers and severity of such incidents will be included in the statistical analysis. All Adverse events (AEs and SAEs) will be recorded on the AE form. Serious adverse events (SAEs) will be reported to the IRB. Toxicity will be assessed using the National Cancer Institute’s Common Toxicity Criteria for Adverse Events, version 5.0 (CTCAEv5) [32].

A collection of adverse events will be conducted at Visit 2 to individuate the AE rate due to neoadjuvant treatment. Neoadjuvant therapy is usually well tolerated, although some patients can require hospital admission for the management of diarrhea or significant late small bowel toxicity, attributable to radiotherapy [33,34,35,36,37].

A following collection of adverse events will be conducted at Visit 3, before LE, to individuate the AE rate due to added treatment (ECT).

### 2.8. Statistic Considerations

#### 2.8.1. Description of Sample Size Calculation and Planned Statistical Analyses

The sample size was determined by the Group-Sequential Superiority approach with a Margin Tests for the Difference of Two Proportions [36] using a complete response rate to the TRG after local excision as an endpoint. Sample size estimation has been carried out by using the superiority hypothesis in two independent parallel sample proportions [38,39,40,41,42]. According to the literature data, neoadjuvant chemo-radiotherapy treatment in LARC results in a complete response of 25% [43,44,45,46,47,48].

The proportion in the treatment group is assumed to be 0.25 under the null hypothesis and 0.50 under the alternative hypothesis. The proportion in the control group is assumed to be 0.25. The significance level of the one-sided test was targeted at 0.10 and the power at 80%.

An interim analysis will be conducted when 28 patients are enrolled (the percent of the sample size accumulated up to the corresponding look will be 40.0%). If a complete response rate to the TRG after local excision is noted in less than 25% of patients in the treatment group and/or feasibility/safety was not demonstrated, the study will be discontinued, otherwise a further 42 patients will be enrolled in the study.

#### 2.8.2. Statistical Analysis

The treatment feasibility/safety of surgery following electrochemotherapy will be assessed considering the number of participants with compromised surgery following electrochemotherapy assessed by the R1 resection rate, circumferential resection margin (CRM) involvement, non mesorectal resection plane, and post-operative complications according to the Clavien-Dindo Classification. A surgical complications rate of 30–40% was reported in the literature for conventional neoadjuvant treatment followed by TME [37]. The evaluation of each arm will be performed at Visit 5 (12 weeks from ECT/8 weeks from LE).

Complete response rate (TRG 1 and 2), disease recurrence rates and percentages will be compared among the two groups. A one-sided Fisher exact test will be performed to test the differences between the groups. Differences will be considered significant at *p* < 0.10. A Chi square test will be performed to assess statistically significant differences among the groups for the categorical variable.

The time interval between the recruitment date and local disease progression will be represented using the time of treatment failure and median and minimum–maximum values will be considered.

The scores obtained by the QOL, pain and satisfaction questionnaires will be evaluated. Any serious adverse events and their intensity, according to the NCI-CTCAE classification (version 5.0), will be registered in the first two days post-procedure.

Mean ± standard deviation will be considered for normally distributed continuous variables while the median and interquartile range (IQR) will be presented for non-normally distributed continuous variables.

For continuous variables, the difference between follow-up values and baseline values will be tested by a 2-sided Student t-test or by a Mann–Whitney test while, for the categorical variables, the McNemar test and the marginal homogeneity test will be use [49,50].

Time to event outcomes will be analyzed using the Kaplan–Meier method and survival curves will be compared using the log-rank test.

Mixed effect regression models will be used to analyze longitudinal data by adjusting for proper covariates.

#### 2.8.3. Interim Analysis

Interim analyses will be fixed after the first step of the study. A conclusive analysis will be performed at the end of the treatment of patients enrolled in the second stage. If a complete response rate to the TRG after local excision is noted in less than 25% of patients in the treatment group, and/or feasibility/safety was not demonstrated, the study will be discontinued. A difference of 20% for both AE rate and surgical complications rate between the two arms will be considered significant enough to discontinue the trial.

## 3. Discussion

The efficiency and safety of ECT have been demonstrated in preclinical and clinical Phase I and I/II studies in patients with melanoma, head and neck squamous cell carcinoma, Merkel cell carcinomas, basal cell carcinoma and adenocarcinoma nodules. The efficacy of ECT has already been proven in skin tumors, and in mucosal and submucosal tissues with high tumor control. Recent studies have demonstrated the feasibility and safety of ECT in the treatment of deep tumors (locally advanced pancreatic cancer, hepatic colorectal metastases, cancerous thrombosis of the portal vein, cholangiocarcinoma hepatocellular carcinoma) [10,11,12,13,14,15,16,17,18,19,20,21,22,23,24,25,26]. In addition, ECT has been used as a neoadjuvant treatment for tumor downstaging before surgical treatment to increase the rate of conservative surgical interventions.

Moreover, colorectal cancer as well as gastric and esophageal tumors have been treated with endoluminal electrodes without adverse events or complications demonstrating a tumor regression (https://clinicaltrials.gov/ct2/show/NCT03040180, accessed on 10 April 2021) [27].

Currently, 45–55% of rectal cancer patients receive preoperative chemo-radiotherapy for LARC. TME is the gold standard in rectal cancer surgery and is comprised of resection of the rectum together with the fatty tissue surrounding the rectum [45]. For LARC, therapy consists of preoperative chemo-radiation therapy (pCRT) or radiotherapy alone followed by TME [51,52,53,54,55,56,57,58,59,60,61,62].

The high morbidity and functional complications associated with TME motivate the search for conservative treatments with sphincter preservation for patients with early rectal cancer at diagnosis and patients with significant tumor regression after pCRT, with the advantage of reducing morbidity and providing a “true” organ-sparing approach [42]. It has also been suggested that a prolongation of the treatment interval might improve overall survival [63,64,65].

Complete pathological response after neoadjuvant therapy in rectal cancer has been shown to be an independent predictor of overall survival and a decrease in local recurrences; up to 30% of patients will achieve a complete or near complete response [46,47,48].

The idea is to use electrochemotherapy in patients with LARC and major clinical response after neoadjuvant therapy, before surgery (local excision of the rectum as the surgical approach used in clinical practice) and assuming an increase in the local control of residual disease and of complete response rate, confirmed by histopathological examination using Tumor Regression Grade. The TRG is a powerful indicator of response to a neoadjuvant treatment in rectal cancer and can measure the effectiveness of neoadjuvant therapy.

ECT has been shown to have limited side effects, a high degree of patient acceptability and acceptable costs. Treatment is performed under local or general anesthesia and does not always require hospitalization. The outcome is to verify the feasibility/safety of electrochemotherapy in this indication and the increase of complete response rate in the treatment group with respect to control group.

## 4. Conclusions

This study is a phase IIb controlled, randomized, clinical trial conducted in order to evaluate the increase in the complete response rate after neoadjuvant treatment followed (experimental group) or not (control group) by ECT in LARC and to spare organ function due to total mesorectal excision (TME).

## Figures and Tables

**Table 1:** Inclusion and Exclusion Criteria.

Inclusion Criteria	Exclusion Criteria
Patients of both sex aged ≥18 years	Age less than 18 years
Histological confirmation of rectal adenocarcinoma	Patients with neoplasia more than 12 cm from the anal margin
Patients undergoing neoadjuvant treatment for locally advanced tumor of the rectum with confirmed major clinical response after neoadjuvant treatment	Patients with stable disease or disease progression after neoadjuvant treatment
Rectal tumor up to 12 cm from the external anal margin	Patients who, for medical reasons, cannot be given bleomycin
Patients must be willing to comply with the study protocol and give their informed written consent	Lesions unsuitable for Electrochemotherapy (ECT) (bony invasion, large vessels infiltration, etc.)
Patients with an Eastern Cooperative Oncology Group (ECOG) status performance <3	Acute lung infection
	Poor lung function symptoms
	Severe coagulation disorders
	Previous allergic reactions to bleomycin
	Previous cumulative bleomycin dose over than 250 mg/m^2^
	Chronic renal dysfunction (creatinine> 150 µmol/L)
	Pregnancy or lactation

**Table 2:** Visits Scheme.

Visit #		1	2	3	4	5	6	7	8	9
**Time**		Month −1 (up to 1 month prior the treatment)	Day 0 ± 3 days	Day 30 ± 3 days	Day 37 ± 3 days	12 ± 1 week	24 weeks ± 1 week	36 ± 1 week	48 ± 1 week	60± 1 week
	**Visit Description**	Restaging, enrolment, randomization	Treatment group: Electrochemotherapy	Local Excision	Histological examination	Follow up visit	Follow up visit	Follow up visit	Follow up visit	Follow up visit
**Type of Assessment**										
**Clinical Evaluation**		X	X			X	X	X	X	X
**Duration of Hospitalisation**										
**CT**		X							X	
**MRI**		X					X		X	
**Histological Examination (TRG and pTNM analysis)**					X					
**Rectal Exploration**						X	X	X	X	X
**Rectoscopy**						X	X	X	X	X
**Colonscopy**									X	
**Pain Evaluation**		X	X	X		X	X	X	X	X
**Blood Samples as per Normal Clinical Practice**			X	X		X	X	X	X	X
**CEA**			X	X		X	X	X	X	X
**Recording of the Drugs for Pain Control and of Concomitant Treatment**				X	X	X	X	X	X	X
**ECOG status**		X								
**EQ-5D-5L Questionnaires**		X	X	X		X	X	X	X	X
**Adverse Events/Complications**			X	X	X	X	X	X	X	X
**Continuing**										
**Visit #**		10	11	12	13	14	15	16	17	18
**Time**		72 ± 1 week	84 ± 1 week	96 ± 1 week	120 ± 1 week	144 ± 1 week	168 ± 1 week	192 ± 1 week	216± 1 week	240 ± 1 week
	**Visit Description**	Follow up visit	Follow up visit	Follow up visit	Follow up visit	Follow up visit	Follow up visit	Follow up visit	Follow up visit	Follow up visit
**Type of assessment**										
**Clinical evaluation**		X	X	X	X	X	X	X	X	X
**Duration of hospitalisation**										
**CT**				X		X		X		X
**MRI**		X		X	X	X	X	X	X	X
**Histological examination (TRG and pTNM analysis)**										
**Rectal exploration**		X	X	X	X	X	X	X	X	X
**Rectoscopy**		X	X	X	X	X	X	X	X	X
**Colonscopy**				X		X		X		X
**Pain evaluation**		X	X	X						
**Blood samples as per normal clinical practice**		X	X	X		X	X	X	X	X
**CEA**		X	X	X	X	X	X	X	X	X
**Recording of the drugs for pain control and of concomitant treatment**		X	X	X	X	X	X	X	X	X
**EQ-5D-5L questionnaires**		X	X	X	optional	optional	optional	optional	optional	optional

Note. CT = Computed Tomography, MRI = Magnetic Resonance Imaging, TRG = Tumor Regression Grade, pTNM = pathological TNM, CEA = carcinoembryonic antigen.

## Data Availability

All data are reported in the manuscript.

## Appendix A. Patient Information Form and Informed Consent

The Operative Unit of Colorectal Surgical Oncology of the I.N.T. “G. Pascale Foundation” as Promoter

Center and Unit ………………………………………………………………………………… conduct a study that aims to evaluate the efficacy of electrochemotherapy with bleomycin in the treatment of locally advanced rectal cancer patients with major clinical response after neoadjuvant treatment.

The title of the study is è: “ORGAN SPARING FOR LOCALLY ADVANCED RECTAL CANCER AFTER NEOADIUVANT TREATMENT FOLLOWED BY ELECTROCHEMIOTHERAPY”.

From the clinical radiological investigations performed for the restaging after neoadjuvant treatment for the pathology of which it is affected, it appears that you possess the clinical characteristics suitable to participate in this study.

We therefore propose that you take part in it and for this purpose, you can obtain detailed information from the doctor in charge or from your collaborators.

However, before you make the decision to accept or refuse to participate, we kindly ask you to read these pages carefully, taking as much time as you need, and to ask for clarifications if you do not understand or need further clarification. Furthermore, if he wishes, he can ask his family members or his doctor for advice before making a decision.

**This research has the general objective of:**

*To evaluate the efficacy (in terms of increase of the complete response rate) of electrochemotherapy with bleomycin in the treatment of locally advanced rectal cancer patients, with a greater clinical response after neoadjuvant treatment.*

The study is a prospective randomized study; therefore it foresees the random insertion in one of the following groups:

- Treatment arm: patients undergoing neoadjuvant treatment for locally advanced rectal cancer with greater clinical response to restaging will undergo Electrochemotherapy (ECT). After 4 weeks after the ECT procedure, the patient will undergo surgical treatment with local excision of residual tissue and histopathological examination.
- Control arm: patients undergoing neoadjuvant treatment for locally advanced rectal cancer with greater clinical response to restaging will undergo surgical treatment with local excision (EL) and histopathological examination.

If you decide to participate, the study provides as follows:

It is a spontaneous multi-center non-profit study that involves the use of the Cliniporator Vitae electrochemotherapy system (IGEA S.p.A.) and the use of appropriate electrodes. The device and the electrodes are CE marked.

Electrochemotherapy (ECT) is the local enhancement, through the electroporation of tumor cells, of chemotherapy antitumor activity of drugs not (ie bleomycin) or poorly permeating (ie cisplatin) cell membrane, with high intrinsic cytotoxicity (sometimes chemotherapy does not easily enter the cell, both healthy and sick).

Bleomycin, the drug that will be used during the study, once inside the cell can carry out its cytotoxic activity irreparably damaging the DNA regardless of the cell type and causing the death of the diseased cell. The efficacy of ECT has been widely documented in solid cutaneous and non-skin neoplasms of different histological origin (melanoma, breast carcinoma, renal adenocarcinoma, squamous cell carcinoma, basalioma, soft tissue sarcoma, salivary gland adenocarcinoma, sarcoma of Kaposi, chondrosarcoma, transitional cell carcinoma of the bladder. ECT is a safe and effective non-thermal tumor ablation modality (does not burn tissue) and is currently standardized for the treatment of skin and subcutaneous lesions. regardless of the histological origin of the tumor.

The following **benefits** can be expected from participating in this study:

- ECT is a technique that uses electrical impulses associated with the administration of bleomycin to cause the death of tumor cells in the target tissue, minimizing possible damage to adjacent tissues.
- ECT has the potential to increase treatment options for patients with head and neck cancer.
- Unlike traditional anti-tumor techniques, which are based on the use of heat to destroy cells such as radiofrequency thermoablation and which can be dependent on tissue conductivity, local interaction of the latter and cooling-mediated perfusion of the fabric, ECT does not generate heat and therefore does not imply any limitation in use.
- This fact, of not using heat to destroy the tumor, will allow us to be more precise and not to damage healthy and important tissues
- Since there is no heat, we will not risk doing unintended distance damage due to local heat transport through vessels (heat sink effect).

Associated with the proposed procedure are the following risks for patients:

- Rare adverse events related to chemotherapy administration (Bleomycin). In patients treated with multiple doses of bleomycin rare serious pulmonary and/or fibrous-type reactions have been found, with the most frequent occurrence of skin and mucous rash; itch; hyperkeratosis; hair and nail loss, alopecia; stomatitis, cutaneous hyperpigmentation (flagellate dermatitis). Other side effects include fever, anorexia, nausea and vomiting, headache. Additional risks related to electroporation in association with bleomycin have not been established.
- Adverse events typical of the surgical act.
- Adverse events related to general anesthesia.

You are free not to participate in the study. In this case, however, you will receive all the standard therapies provided for your condition, without any penalty, according to good clinical practice.

There may be alternative to treatments currently in use for the treatment of your condition such as oncological chemotherapy treatments or radiotherapy.

For more information on traditional techniques and its therapeutic options, we invite you to consult the doctor responsible for the trial.

Participation in the study could involve some risks related to the drug administration or to the treatment itself.

Since ECT is a recent technique, there may be potential risks and side effects that are currently unknown. A risk analysis was conducted to identify potential hazards associated with the device and the clinical risks associated with the procedure as well as systems to minimize these risks.

We also inform you that the procedure is carried out in conscious sedation or general anesthesia, with the related risks involved.

If you are a woman of childbearing age, you must not become pregnant during the trial period. No contraceptive method used constitutes a contraindication to participation in the study.

If you cannot rule out the possibility of pregnancy during the study, you will not be able to participate in this trial to avoid harmful effects on the embryo/fetus.

Your pregnancy status will be checked first by enrolment via urine Beta-hCG (pregnancy test or urinary beta-HCG) or blood (plasma beta-hCG).

Your participation in this research program is voluntary and you can withdraw from the study at any time.

In the same way, the trial can be interrupted if the doctor does not notice a benefit or if unwanted or other effects have occurred.

In these cases, you will be promptly informed of further valid treatments for your and may discuss them with your doctor.

If data that could influence the decision to continue the study in question become available, you will be promptly informed.

The proposed study protocol was drafted in compliance with the European Union Good Clinical Practice Rules and the current revision of the Helsinki Declaration and was approved by the Ethics Committee of this Company.

We underline that all the patients enrolled in the trial proposed to you, as well as the hospital and the doctors involved in the experimentation will be covered by an adequate insurance policy stipulated ad hoc, which guarantees the coverage of civil liability damages resulting from the experimentation itself.

The processing of personal data of all patients enrolled in the trial proposed to you must be developed in full compliance with the rules contained in the GDPR EU Regulation n. 2016/679 (Code regarding the protection of personal data).

Authorized personnel of the Foundation, the Ministry of Health and the manufacturer of the device under study [IGEA SpA] may need to access the information of the individual subjects, and therefore of their name, but are bound by confidentiality rules not to disclose to others your identity.

At the time of publication of the results of the study or of their dissemination in the congress, there will be no information that reveals your identity.

Your participation in this study takes place on a VOLUNTARY basis. Failure to participate will have no consequences on your relationship with the Foundation, or on your right to receive care or other services provided by this institution. If you decide to participate, you are free to withdraw your consent and withdraw from the study at any time, without any consequences on the assistance you will receive from this institution in the future.

The experimenter may decide to withdraw you from the study if necessary. If you observe one or more of the following side effects or if you become ill during the study, you may have to abandon it even if you want to continue. The investigator will decide on this and let you know if it is possible for you to continue to participate in the study. The decision may be aimed at safeguarding your health and safety, or required by the study protocol, which may indicate that individuals who develop particular pathologies must be excluded.

During the study, you will be notified of any new findings (positive or negative) that may change the risks or benefits of your participation in the research, or new alternatives that could change your mind about continuing it. If new elements are provided, you will be asked to renew your consent to continue your participation in the study.

You have the right to withdraw your consent at any time and stop participating in the study without being penalized. By participating in the study, you do not give up any of your legal rights to assistance and therapy.

Neither you nor your eventual health insurance will have to face medical expenses (examinations, procedures, devices) for your participation in this study.

Given the above, I the undersigned …………………………………..………… born to ………………………….………… I declare to have been invited to read very carefully what is reported in this paper which corresponds, moreover, to what has been amply explained to me, by Prof/Dr. ……………………………………………………………. I also declare that I have understood the meaning of what I was exposed to, that I do not need further clarification and therefore knowingly - I agree □ - I do not agree □ to submit to the proposed procedure; Date……………………………. The Patient signature The Doctor _______________________ _______________________
